# Natural Products; from the Laboratory to Clinical Practice

**DOI:** 10.3390/molecules28073184

**Published:** 2023-04-03

**Authors:** Fawzy Elbarbry

**Affiliations:** School of Pharmacy, Pacific University Oregon, Hillsboro, OR 97123, USA; fawzy.elbarbry@pacificu.edu

It has been such a great honor to serve as the Guest Editor for this Special Issue, “Exploration on Pharmacokinetics and Pharmacodynamics of Natural Molecules: Current Status and Future Perspectives”. I have been very interested in studying small natural molecules, especially with regard to their impact on organ systems and potential uses as therapeutic aids for the prevention and/or treatment of chronic illnesses; therefore, I was very excited to accept this role despite its anticipated challenging nature. In the early 1990s, the research and discovery efforts devoted to many pharmaceuticals were focused on combinatorial chemistry and high-throughput screening to generate and identify new drugs; however, this switch did not yield the expected returns in terms of new drug candidates, and might in fact have led to the current paucity of new drug candidates in the development pipeline. Therefore, researchers directed their attention to the natural product “*toolbox*” in an effort to develop safe, effective, and cost-effective therapeutic approaches. Searching PubMed with the search terms “Natural Products” AND “Pharmacokinetics” AND “Pharmacodynamics”, yielded more than 20,000 articles.

For this Special Issue, authors were asked to share their latest findings obtained whilst exploring the physicochemical, pharmacokinetic, and pharmacodynamic properties of promising natural products. Several high-quality submissions were received, and only nine were published after an extensive peer review process. These articles signify a great example of how natural products and phytochemicals can play a key role in the biomedical field.

Two articles demonstrated the blood-pressure-lowering effect of small natural molecules, namely catechin and honokiol, through a novel mechanism involving arachidonic acid metabolism [1,2].

One study extensively investigated the pharmacokinetics of the multiple components of Gelsemium elegans after feeding it to pigs in medicated feed [3]. This study provides an important reference with which to explain the efficacy and safety of G. elegans in pigs, especially since it is commonly used as a feed additive. 

Another study in this Special Issue examined the pharmacokinetic properties of a natural macrolide antibiotic, azalomycin F [4]. During this era of an alarming increase in antimicrobial resistance, the exploration of new antimicrobials derived from nature is desperately needed. This study demonstrated the poor oral bioavailability of azalomycin F; therefore, an intravenous route must be used in treating systemic infections, while oral administration is limited to treating local gastrointestinal diseases. 

The development of sensitive and accurate analytical techniques is pivotal in biomedical research. This Special Issue provides two articles that demonstrate the use of liquid chromatography-tandem mass spectrometry (LC-MS/MS) in studying the pharmacokinetic characteristics of the components of Withania somnifera, a traditional Indian herb [5], and AWRK6, a synthesized peptide developed based on the naturally occurring peptide dybowskin-2CDYa, which was discovered in frog skin [6]. 

Two articles in this Special Issue investigate the antioxidant and anti-inflammatory effects of select phytochemicals, namely Ficus religiosa [7] and green apple flavonols [8]. 

Finally, this Special Issue houses a very thorough and comprehensive literature review article that examines the underlying mechanisms through which several natural products can alleviate the inflammation associated with chronic illnesses [9].

Again, it was a wonderful journey leading this Special Issue, and I was able to enjoy reading great as well as high-quality research studies and network with brilliant minds from all over the Earth. 
